# Multiple Sklerose: Infektionsprophylaxe und Impfungen

**DOI:** 10.1007/s00739-021-00703-6

**Published:** 2021-01-28

**Authors:** Thomas Seifert-Held

**Affiliations:** Univ.-Klinik für Neurologie, Medizinische Universität Graz, Auenbruggerplatz 22, 8036 Graz, Österreich

**Keywords:** Multiple Sklerose, Krankheitsmodifizierende Therapie, Infektionen, Impfungen, SARS-CoV‑2, Multiple sclerosis, Disease-modifying therapies, Infection, Vaccination, SARS-CoV‑2

## Abstract

Hochwirksame krankheitsmodifizierende Therapien der multiplen Sklerose sind mit einem allgemein höheren Risiko für Infektionserkrankungen verbunden. Darüber hinaus bestehen substanzspezifische Risiken. Unter Anti-CD20-Therapien ist die Reaktivierung einer Hepatitis-B-Infektion möglich. Die Reaktivierung einer Tuberkulose ist vor der Anwendung von Teriflunomid, Cladribin und Alemtuzumab zu berücksichtigen. Zur Risikostratifizierung des Auftretens einer progressiven multifokalen Leukenzephalopathie unter Therapie mit Natalizumab ist der Anti-JCV-Antikörperindex etabliert. Eine Vorbeugung von Varizella-zoster-Virus- (VZV-)Infektionen ist für eine Therapie mit Fingolimod, Cladribin und Alemtuzumab erforderlich. Vor Beginn einer hochwirksamen krankheitsmodifizierenden Therapie sollen ausständige Impfungen verabreicht werden, dabei müssen Lebendimpfungen, wie z. B. gegen VZV, mindestens 4 Wochen vor Therapiebeginn appliziert werden. Unter Therapie können nur Totimpfstoffe verwendet werden, deren Impferfolg reduziert ist. In der aktuellen COVID-19-Pandemie gehören Patient(inn)en mit hochwirksamen krankheitsmodifizierenden Therapien zur Risikogruppe. Die aktuell (Stand Januar 2021) gegen SARS-CoV‑2 zugelassenen Impfstoffe sind unter diesen Therapien anwendbar.

## Abstract (en)

In multiple sclerosis, highly effective disease-modifying therapies are associated with the risk of infectious diseases, and substance-specific aspects must be considered. Hepatitis B reactivation may occur with anti-CD20 treatments. A screening for latent tuberculosis is recommended before treatment with teriflunomide, cladribine and alemtuzumab. To stratify for the risk of progressive multifocal leukoencephalopathy with natalizumab treatment, the anti-JCV-antibody index is established. Prevention of varicella zoster virus (VZV) infection is recommended prior to treatment with fingolimod, cladribine and alemtuzumab. Vaccinations should be completed before the start of a highly effective disease-modifying treatment. Live-attenuated vaccines, e.g. the VZV vaccine, have to be administered at least 4 weeks before and should not be given during treatment. Vaccine efficacy is reduced when given with highly effective disease-modifying treatments. In the current COVID-19 (coronavirus disease 2019) pandemic, highly effective disease-modifying therapies are considered to confer an increased risk. The currently (January 2021) approved SARS-CoV‑2 (severe acute respiratory syndrome coronavirus type 2) vaccines can be administered with concomitant disease-modifying therapies.

## Infektionen unter krankheitsmodifizierenden Therapien

Untersuchungen in großen Erkrankungsregistern haben gezeigt, dass Patient(inn)en mit multipler Sklerose (MS) häufiger von Infektionserkrankungen betroffen sind als die Gesamtbevölkerung und hierfür auch häufiger stationär behandelt werden müssen. Unterschiede im Infektionsrisiko zeigen sich zwischen den verwendeten Medikamenten. Hochwirksame krankheitsmodifizierende Therapien sind mit einem höheren Risiko für Infektionen verbunden.

Die Reaktivierung einer Hepatitis-B-Infektion ist ein Risiko von Anti-CD20-Therapien (Rituximab, Ocrelizumab). Vor Beginn soll daher der Hepatitis-Antikörper-Status bestimmt werden. Zeigt dieser eine abgelaufene Hepatitis-B-Infektion an, dann muss eine virusstatische Therapie während und bis 12 Monate nach Ende der Anti-CD20-Therapie eingenommen werden. Von der Anwendungserfahrung mit dem Prodrug Leflunomid in der Rheumatologie ergibt sich für Teriflunomid ein Risiko für die Reaktivierung einer Tuberkulose. Bei den über 2000 mit Teriflunomid in den Studien TEMSO, TOWER, TOPIC und TENERE behandelten Patienten trat eine Tuberkulose in 3 Fällen auf. Daher ist vor Therapiebeginn ein Interferon‑γ Release Assay (IGRA) erforderlich. Ein IGRA wird auch vor Einleitung einer Therapie mit Cladribin oder Alemtuzumab empfohlen. Die Reaktivierung einer Tuberkulose sollte aber künftig auch vor Beginn anderer krankheitsmodifizierender Therapien bedacht werden. In einer Untersuchung im Jahr 2017 an zwei großen deutschen MS-Zentren fand sich eine latente Tuberkuloseinfektion bei 4 % aller Patienten.

Hochwirksame Therapien sind mit einem höheren Risiko für Infektionen verbunden

Das Risiko für das Auftreten einer progressiven multifokalen Leukenzephalopathie (PML) unter einer Therapie mit Natalizumab schränkt die Anwendung dieses Medikaments ein. Die Gesamtinzidenz der PML wird aktuell mit 3,99 auf 1000 mit Natalizumab behandelte Patienten angegeben. Zur Risikostratifizierung wird der Anti-JCV-Antikörperindex (Stratify JCV™-Test, Fa. Unilabs, Kopenhagen, Dänemark) verwendet. Hier handelt es sich nicht um einen Antikörpertiter, wie wir ihn aus der Infektionsserologie kennen, sondern um eine Ratio der Anti-JCV-Antikörper aus Patientenserum und der Anti-JCV-Antikörper einer Kalibratorlösung. Werte über 0,4 gelten als positiv. Bei negativem Anti-JCV-Antikörperindex kann eine Therapie mit Natalizumab begonnen werden, Indexbestimmungen werden dann alle 6 Monate und MR-Verlaufskontrollen alle 12 Monate empfohlen. Bei Serokonversion unter Natalizumab-Therapie auf einen Index < 0,9 ist ein Weiterführen möglich. Bei Serokonversion auf Indexwerte > 0,9 soll Natalizumab nach max. 24 Monaten Gesamttherapiedauer beendet werden. Der Anti-JCV-Antikörperindex ist nur zur Risikostratifizierung einer PML unter Therapie mit Natalizumab validiert.

Im Vergleich zu Natalizumab sehr viel geringer ist das Risiko für eine PML unter anderen krankheitsmodifizierenden Therapien. Mit Dimethylfumarat trat bisher bei 11 von 475.000 Behandelten eine PML auf. Laut aktueller Sicherheitsinformation soll bei einer Lymphozytenzahl < 0,5 × 10^9^/l eine Therapie mit Dimethylfumarat nicht begonnen bzw. beendet werden, wenn dieser Wert für mehr als 6 Monate unterschritten wird.

In einer Zulassungsstudie für Fingolimod (TRANSFORMS) sind zwei Patient(inn)en an Herpesenzephalitis bzw. disseminiertem Herpes zoster verstorben. In den Phase-II- und -III-Studien mit Fingolimod war jedoch die Inzidenz von Varizella-zoster-Virus- (VZV-)Infektionen nicht höher als in den Phase-II- und -III-Studien mit β‑Interferonen, Dimethylfumarat oder Natalizumab. Vor Einleitung einer Therapie mit Fingolimod soll der Antikörperstatus gegen VZV bestimmt und bei fehlender Immunität geimpft werden. Dies gilt auch, wenn eine Therapie mit Cladribin oder Alemtuzumab geplant ist, für letzteres ist auch eine virusstatische Prophylaxe zumindest in den ersten 4 Wochen nach Applikation erforderlich. Für Cladribin wird eine virusstatische Prophylaxe bei Lymphozytenzahlen < 0,2 × 10^9^/l empfohlen.

## Impfungen unter krankheitsmodifizierenden Therapien

Allgemein empfohlene Impfungen sind nicht mit der Diagnose bzw. Schüben der MS assoziiert. Vermutungen über einen Zusammenhang der Entwicklung einer MS nach Hepatitis-B-Impfungen in Frankreich in den 1990er-Jahren wurden durch große Studien, publiziert Anfang der 2000er-Jahre, widerlegt. Hier zeigte sich kein Zusammenhang der Entwicklung einer MS mit stattgehabten Impfungen gegen Hepatitis B, Tetanus, Influenza, Masern, Mumps und Röteln. Dies wurde in einer 2019 publizierten großen Studie aus Bayern bestätigt. Hier fand sich auch für Impfungen gegen Varizellen, Pertussis, Haemophilus influenzae B, Pneumokokken, Meningokokken, humanes Papillomvirus und Frühsommer-Meningoenzephalitis (FSME) keine Assoziation mit der späteren Diagnose einer MS. Erkrankungsschübe werden nicht durch Impfungen ausgelöst. Dies wurde untersucht für Impfungen gegen Hepatitis B, Tetanus (auch in Kombination mit Diphtherie und/oder Poliomyelitis), Influenza und FSME. Eine Ausnahme ist die Gelbfieber-Impfung, eine Reiseimpfung mit einem Lebendimpfstoff. Hier zeigte sich in einer Fallserie mit 7 MS-Patient(inn)en eine erhöhte Schubrate nach Impfung.

Allgemein empfohlene Impfungen sind nicht mit Schüben der MS assoziiert

Unter hochwirksamer krankheitsmodifizierender Therapie sollen keine Lebendimpfstoffe appliziert werden. Wenn eine Auffrischungsimpfung mit Lebendimpfstoffen (Masern, Mumps, Röteln, VZV) erforderlich ist, dann muss diese mindestens 4 Wochen vor Beginn der Therapie erfolgen. Vor Therapiebeginn erlangte Impftiter gegen Mumps, Röteln und VZV bleiben für mindestens 2 Jahre unter einer Anti-CD20-Therapie unverändert. Längerfristig können hier jedoch die Impftiter im Vergleich zu Unbehandelten früher abfallen. So ist die Halbwertzeit für Anti-Tetanus-IgG unter Rituximab-Therapie mit 5,5 Jahren nur halb so lang wie in der Allgemeinbevölkerung.

Bei bestehender krankheitsmodifizierender Therapie ist der Impferfolg, d. h. die Höhe des Impftiters, von der verwendeten Substanz abhängig. Beta‑Interferone, Teriflunomid und Dimethylfumarat beeinflussen den Impferfolg nicht. Unter Therapie mit Fingolimod sind Anti-Tetanus-Impftiter vergleichbar zu Kontrollpersonen, Anti-Influenza-Titer jedoch reduziert. Die Impfantwort auf Influenza ist auch unter einer Therapie mit Siponimod, Glatiramerazetat und Natalizumab verringert. Impftiter gegen Tetanus, Pneumokokken und Influenza sind unter Anti-CD20-Therapien reduziert, wobei jedoch für einige untersuchte Influenza-Impfstämme unter Therapie mit Ocrelizumab ein zu Kontrollpersonen vergleichbarer Impftiter erreicht werden konnte. Die Durchführung von Impfungen unter bestehender Anti-CD20-Therapie wird frühestens 3 Monate nach der letzten und mindestens 4 Wochen vor der nächsten Applikation empfohlen.

Unter hochwirksamer Therapie sollen keine Lebendimpfstoffe appliziert werden

Bisherige Studien über den Impferfolg unter krankheitsmodifizierender Therapie beschreiben hauptsächlich Antikörpertiter, kaum jedoch Mechanismen der zellulären Immunität. Antikörpertiter können ein Menschenleben lang erhalten bleiben. So waren bei Personen, die die Spanische Grippe im Jahr 1918 erlebt haben, noch 90 Jahre später neutralisierende Antikörper und antigenspezifische zirkulierende B‑Gedächtniszellen nachweisbar. Unter Anti-CD20-Therapie ist jedoch langfristig ein Abfall vorbestehender Impftiter zu erwarten. Problematisch hierbei ist, dass Auffrischungsimpfungen unter Anti-CD20-Therapien geringere Impfantworten zeigen. Die Analyse einer Open-Label-Nachfolgebeobachtung einer Phase-II-Studie mit Ocrelizumab ergab, dass zirkulierende B‑Gedächtniszellen bis zumindest 18 Monate nach der letzten Applikation des Medikamentes supprimiert bleiben, was entscheidend ist für die Unterdrückung neuer entzündlich-demyelinisierender Aktivität. Dagegen kommt es bereits 12 Monate nach der letzten Applikation von Ocrelizumab zur Repopulation naiver B‑Zellen auf 60 % des Wertes vor Therapiebeginn. Hier, im Zeitraum von 12 bis 18 Monaten nach letzter Applikation, wird von manchen Autoren ein Zeitfenster zur Durchführung von Impfungen zum Erreichen eines ausreichenden Impftiters bei noch bestehendem Schutz vor entzündlich-demyelinisierender Aktivität gesehen. Für ein solches über 6 Monate hinaus verlängertes Dosierungsintervall von Ocrelizumab gibt es jedoch gegenwärtig keine Studien und keine Zulassung.

## Impfungen gegen SARS-CoV-2

Nach Infektion mit SARS-CoV‑2 sind im peripheren Blut B‑Gedächtniszellen spezifisch für das Spike-Protein und die rezeptorbindende Domäne in einem Beobachtungszeitraum von zumindest 5 Monaten nachweisbar. IgG-Antikörper gegen das Spike-Protein und die rezeptorbindende Domäne von SARS-CoV‑2 finden sich bei fast allen Erkrankten, jedoch mit sehr hoher Heterogenität der Antikörpertiter. Die beiden zuerst in der EU zugelassenen Impfstoffen gegen SARS-CoV‑2, BNT162b2 (Comirnaty®; Fa. Biontech/Pfizer) und mRNA-1273 (COVID-19 Vaccine Moderna), generieren bei Geimpften höhere Titer neutralisierender Antikörper als sie bei von COVID-19 Genesenen gefunden werden. Beide Impfstoffe basieren auf modifizierter mRNA, verkapselt in Lipid-Nanopartikel. Die beiden genannten Impfstoffe sind die ersten zugelassenen Impfstoffe überhaupt mit dieser Technologie. Die im Impfstoff enthaltene mRNA kodiert das SARS-CoV-2-Spike-Protein im Zustand vor der Fusion mit seinem Rezeptor. Die mRNA im Impfstoff wird stabilisiert und ihre Translation gefördert durch Modifikationen an ihren 5’- und 3’-Enden. Die injizierte mRNA amplifiziert sich nicht und wird nicht in das Genom eingebaut. Der Protein-Synthese-Apparat der Zellen am Injektionsort, hauptsächlich Muskelzellen und dendritische Zellen, wird genutzt, um das gewünschte Protein, hier das SARS-CoV-2-Spike-Protein, zu synthetisieren. Dieses wird dann auf der Zelloberfläche präsentiert. Es resultiert eine lokale immunologische Reaktion, die dann in die lokalen Lymphknoten und weiter systemisch propagiert wird. In der Folge kommt es zur Aktivierung zytotoxischer T‑Zellen und zur Differenzierung von B‑Zellen zu antikörpersezernierenden Plasmazellen. Für die beiden mRNA-basierten Impfstoffe wurde ihre Wirksamkeit auch im höheren Lebensalter gezeigt. Aktuell laufen Untersuchungen über ihre Wirksamkeit bei Immunsupprimierten.

Adenovirale Vektoren sind eine weitere Technologie, die in der Entwicklung von Impfstoffen gegen SARS-CoV‑2 zur Anwendung kommt. Darunter sind ChAdOx1 nCoV-19 (COVID-19 Vaccine AstraZeneca) und Ad26.COV2.S (Fa. Janssen-Cilag/Johnson & Johnson) im Zulassungsprozess in der EU am weitesten fortgeschritten. Hier wurde in die DNA eines replikationsgehemmten Adenovirus die genetische Information des SARS-CoV-2-Spike-Proteins eingefügt. Auch hier wird der Protein-Synthese-Apparat der Zellen am Injektionsort genutzt, um das SARS-CoV-2-Spike-Protein zu synthetisieren und eine Immunantwort zu generieren.

Alle genannten Impfstoffe sind grundsätzlich zur Impfung immunsupprimierter Patient(inn)en geeignet, da es sich nicht um Lebendimpfstoffe handelt.

Für MS-Patient(inn)en mit hochwirksamen krankheitsmodifizierenden Therapien wird ein erhöhtes Risiko für einen schweren Verlauf einer COVID-19-Infektion angenommen. Substanzspezifische Aussagen sind aktuell (Stand Januar 2021) jedoch noch nicht zuverlässig möglich. Für eine Impfung gegen SARS-CoV‑2 wird sowohl Patient(inn)en mit Autoimmunerkrankungen als auch immunsupprimierten Patient(inn)en laut Empfehlung des Nationalen Impfgremiums die Priorität „erhöht“ zugeordnet, die dritthöchste von 7 Prioritätsstufen.

## Fazit für die Praxis

- Hochwirksame krankheitsmodifizierende Therapien der Multiplen Sklerose (MS) sind mit substanzspezifischen Risiken für Infektionserkrankungen verbunden.
- Es gibt keinen Zusammenhang der Entwicklung einer MS oder des Auftretens von Erkrankungsschüben mit allgemein empfohlenen Impfungen.
- Unter einer bestehenden krankheitsmodifizierenden Therapie sollen keine Lebendimpfstoffe, sondern nur Totimpfstoffe angewendet werden.
- Für eine Impfung gegen SARS-CoV‑2 wird Patient(inn)en mit hochwirksamen krankheitsmodifizierenden Therapien laut Empfehlung des Nationalen Impfgremiums die dritthöchste Priorität zugeordnet.

